# Cutaneous and pulmonary manifestations of sarcoidosis triggered by coronavirus disease 2019 infection

**DOI:** 10.1590/0037-8682-0647-2021

**Published:** 2022-04-08

**Authors:** Felipe Tavares Rodrigues, Renata Miguel Quirino, Alexandre Carlos Gripp

**Affiliations:** 1 Universidade do Estado do Rio de Janeiro, Programa de Pós-Graduação em Dermatologia, Rio de Janeiro, RJ, Brasil.; 2 Universidade do Estado do Rio de Janeiro, Hospital Universitário Pedro Ernesto, Departamento de Dermatologia, Rio de Janeiro, RJ, Brasil.

The coronavirus disease 2019 (COVID-19) can cause dermatological manifestations such as urticarial rash, confluent erythematous/maculopapular/morbilliform rash, papulovesicular exanthema, chilblain-like acral pattern, livedo reticularis/racemosa-like patterns, and purpuric “vasculitic” patterns^1^.

Herein, we describe a case of sarcoidosis with exuberant cutaneous and pulmonary manifestations after recovery from COVID-19 infection. In the literature, only two cases of granulomatous sarcoid-like reaction have been described, with few lesions and no pulmonary involvement of the disease^2^. 

**FIGURE 1:**
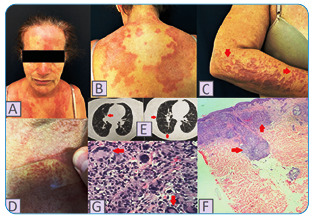
**(A)** Erythematous plaques on the face and chest. **(B)** Erythematous plaques with a symmetrical arrangement on the back. **(C)** Some lesions with annular dispositions on the patient’s arm (red arrows). **(D)** The pressure from the glass slide application eliminates the characteristic erythema, revealing a yellow-brown granulomatous appearance resembling apple jelly. **(E)** Chest tomography performed six months after presenting with flu-like symptoms, demonstrating ground-glass infiltrates affecting 25-50% of the lung area (red arrows). **(F)** Skin biopsy showing rectification of the epidermis and a mononuclear infiltrate in the dermis, with formation of non-necrotizing granulomas (red arrows). HE, increased 10x. **(G)** Visual of the naked sarcoidal granulomas in more detail, with the presence of Langhans cells (red arrows) and without the presence of caseous necrosis. **HE:** increased 40x.

A 57-year-old woman with no previous comorbidities was admitted to the dermatology department with erythematous, symmetrical, non-pruritic papules and plaques; some had an annular disposition over six months of evolution (Figures 1A-D). The patient had a persistent dry cough for three months. The first lesions appeared about 20 days after the patient presented with symptoms of flu syndrome (fever, runny nose, headache, fatigue, and anophthalmia), and they did not require hospitalization. A nasal swab test with polymerase chain reaction confirmed a SARS-CoV-2 infection. The diagnosis of sarcoidosis was confirmed via skin biopsy (Figure 1F and 1G). Chest computed tomography (CT) revealed pulmonary involvement (Figure 1E). The tuberculin test result was negative, and the patients’ serum calcium levels were slightly above the normal limits. We started treatment with 60 mg prednisone plus 100 mg azathioprine per day, which improved the patient’s symptoms. 

There may be an intrinsic relationship between viral infections and sarcoidosis development in genetically susceptible hosts^3^.
